# *In silico* prediction of siRNA ionizable-lipid nanoparticles *In vivo* efficacy: Machine learning modeling based on formulation and molecular descriptors

**DOI:** 10.3389/fmolb.2022.1042720

**Published:** 2022-12-21

**Authors:** Abdelkader A. Metwally, Amira A. Nayel, Rania M. Hathout

**Affiliations:** ^1^ Department of Pharmaceutics, Faculty of Pharmacy, Health Sciences Center, Kuwait University, Kuwait City, Kuwait; ^2^ Department of Pharmaceutics and Industrial Pharmacy, Faculty of Pharmacy, Ain Shams University, Cairo, Egypt; ^3^ Clinical Pharmacy Department, Alexandria Ophthalmology Hospital, Alexandria, Egypt; ^4^ Department of Clinical Pharmacy and Pharmacy Practice, Faculty of Pharmacy, Alexandria University, Alexandria, Egypt

**Keywords:** siRNA, ionizable lipids, nanoparticles, *in vivo*, QSAR, machine learning

## Abstract

*In silico* prediction of the *in vivo* efficacy of siRNA ionizable-lipid nanoparticles is desirable as it can save time and resources dedicated to wet-lab experimentation. This study aims to computationally predict siRNA nanoparticles *in vivo* efficacy. A data set containing 120 entries was prepared by combining molecular descriptors of the ionizable lipids together with two nanoparticles formulation characteristics. Input descriptor combinations were selected by an evolutionary algorithm. Artificial neural networks, support vector machines and partial least squares regression were used for QSAR modeling. Depending on how the data set is split, two training sets and two external validation sets were prepared. Training and validation sets contained 90 and 30 entries respectively. The results showed the successful predictions of validation set log (siRNA dose) with R_val_^2^= 0.86–0.89 and 0.75–80 for validation sets one and two, respectively. Artificial neural networks resulted in the best R_val_^2^ for both validation sets. For predictions that have high bias, improvement of R_val_^2^ from 0.47 to 0.96 was achieved by selecting the training set lipids lying within the applicability domain. In conclusion, *in vivo* performance of siRNA nanoparticles was successfully predicted by combining cheminformatics with machine learning techniques.

## 1 Introduction

The process of developing short interfering RNA (siRNA) lipid nanoparticles is lengthy and time consuming because it involves the initial chemical synthesis of a usually large number of ionizable lipids and lipid-like molecules (Jayaraman et al., 2012; Sato et al., 2019; Molla et al., 2020), the formulation of siRNA nanoparticles and the subsequent *in vitro* and *in vivo* evaluation of these nanoparticles, in an attempt to find the best ionizable lipid that is suitable for clinical use in terms of efficacy and safety. Alnylam’s small interfering RNA (siRNA) stable nucleic acid lipid nanoparticles, currently marketed as Onpattro™ (Patisiran), obtained FDA approval in 2018. This was followed by FDA approval of Alnylam’s Givosiran™ and Lumasiran™ in 2019 and 2020 respectively (Zhang et al., 2021).

Gene silencing by double-stranded RNA (dsRNA) was reported by Fire and Mello in *Caenorhabditis elegans* (Fire et al., 1998) and later siRNA duplexes of length 21-22 nucleotides proved to promote post-transcriptional gene silencing in mammalian cells (Elbashir et al., 2001). Since then, the potential of siRNA as a therapeutic macromolecule against many diseases has been investigated, with more than 40 siRNA based therapies already reaching phases 2, 3 or 4 of clinical trials (Titze-de-Almeida et al., 2017; Dong et al., 2019; ClinicalTrials.gov, 2020). The major barriers against the successful employment of therapeutic siRNA include the lack of stability of the siRNA duplex, the immune response mediated by Toll-like receptors, the rapid renal clearance of naked siRNA, and the difficulty of the intracellular delivery of unmodified siRNA due to its large size and the large number of negative charges on its back-bone (Whitehead et al., 2009; Dowdy, 2017).

One method to overcome the barriers of siRNA delivery is to formulate it as siRNA ionizable lipid nanocomplexes (lipoplexes) or lipidic nanoparticles (Metwally et al., 2012a; Metwally et al., 2012b; Cullis & Hope, 2017; Paunovska et al., 2018). These nanoparticles are multicomponent and may also contain helper lipids, PEG-lipids and phospholipids. An ideal delivery system should ensure response reproducibility, non-immunogenicity, good payload and ease of manufacturing (Cullis & Hope, 2017).

Lipidic nanocarriers for siRNA include liposomes, nanoemulsions, solid lipid nanoparticles, nanostructured lipid carriers, micelles, and liquid crystalline nanoparticles. Since the nature and ratio of ionizable lipids affects the performance of lipid-nucleic acid complexes, the structure of lipid-based self-assembled nucleic acids delivery systems was investigated and was found to tune the supramolecular organization of the complexes (Angelov et al., 2017).

The process of preparing siRNA lipoplexes and nanoparticles involves many steps: the synthesis of the ionizable lipids, their purification and characterization, then the process of preparing the nanoparticles including determining the siRNA to cationic lipid ratio, the cationic lipid to helper lipid (if any) ratio, and nanoparticles characterization in terms of their size, zeta potential, pK_a_, stability and *in vivo* evaluation of their safety and silencing efficacy. All of these steps require time and resources and indeed if the *in vivo* efficacy, as measured by either the siRNA dose or knockdown efficiency, could be predicted within reasonable accuracy by using computational means, the process of developing siRNA nanomedicines would be vastly improved in terms of time and costs. Therefore, it is important to attempt to predict the *in vivo* efficacy of siRNA cationic lipid nanoparticles by using machine learning techniques. These techniques can be generally classified into two main groups: supervised and unsupervised learning methods. Supervised learning is used in tasks such as regression and classification, i.e., when there is a dependent variable and one or more independent variables.

In order to extract chemical information from the structures of the molecules under investigation, molecular descriptors, which are important cheminformatics tools, are employed to carry out this task (Hathout et al., 2018; Hathout et al., 2020a). Molecular descriptors are numerical values resulting from either an experimental procedure or from a set of mathematical and/or logical algorithms that are performed on chemical structures (Todeschini & Consonni, 2008). The descriptors can be generally classified as 0D and 1D, when only molecular formula or constitutional properties of a molecule are considered, while 2D descriptors are calculated based on topological properties of a molecule and 3D descriptors depend on geometrical properties of a molecule. Further classifications include 2.5D chiral descriptors and descriptors with more than three dimensions (Consonni & Todeschini, 2010; Valdés-Martiní et al., 2017). Molecular descriptors have been used as predictors of the self-assembly of drug molecules into nanoparticles (Shamay et al., 2018), to model drug binding kinetics (De Benedetti & Fanelli, 2018), in QSAR modeling (Kausar & Falcao, 2018) and in target identification (Reker et al., 2014). Molecular descriptors were also used to successfully predict the binding energy between drug molecules and their nanocarriers and hence predict drug loading onto lipidic and polymeric nanoparticles (Metwally & Hathout, 2015).

Previous QSAR studies on nanoparticles have mostly addressed predicting the cellular uptake and toxicological properties of inorganic nanoparticles, with either unmodified or modified surfaces (Liu et al., 2015; Basant & Gupta, 2017; Wang et al., 2017), however, developing QSAR models for predicting siRNA *in vivo* efficacy has not been achieved before.

**FIGURE 1:**
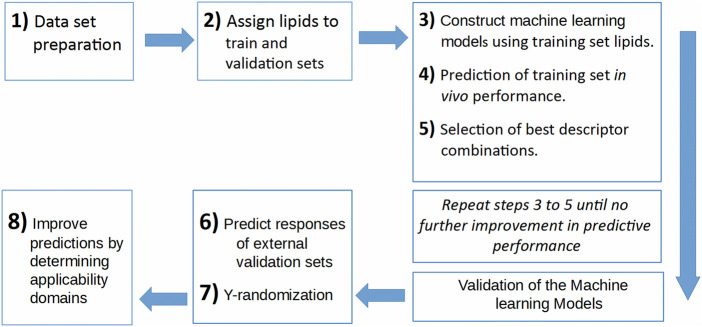
The workflow of the predictive model building process.

In the current work, a data set is prepared using five publications (Jayaraman et al., 2012; Alabi et al., 2013; Kumar et al., 2014; Whitehead et al., 2014; Rajappan et al., 2020). This data set contains the 1D and 2D descriptors of ionizable lipids together with the formulation descriptors (PEG mol%) and the percentage knockdown resulting from a specific siRNA dose. The siRNA nanoparticles *in vivo* efficacy when formulated with these ionizable lipids was included as the response variable; logarithm of the siRNA dose resulting in a specific knockdown percent of the target gene. The data set is split into training and validation sets, where the training set is used to construct the machine learning models, and the validation set is used as an external test set that is used only to evaluate the predictive models constructed by modeling the training set. An evolutionary algorithm is used to select the best descriptor combinations and is combined with three machine learning techniques; ANN, SVM and PLS regression, to build the predictive models. The performance of the predictive models using the three machine learning techniques and the quality of predictions and how to improve them is presented and discussed. Figure 1 shows the workflow of the modeling and evaluation process. A graphical abstract image is provided in Supplementary Files.

## 2 Materials and methods

### 2.1 Data set preparation

#### 2.1.1 Data selection from available literature

For preparing the data set, five publications**(** Jayaraman et al., 2012; Alabi et al., 2013; Kumar et al., 2014; Whitehead et al., 2014; Rajappan et al., 2020) were retrieved after carrying out online search using PUBMED and Google Scholar servers, where all of them fulfilled the following requirements: siRNA is delivered by means of ionizable lipids, siRNA *in vivo* performance is evaluated *in vivo* against factor FVII expression, all nanoparticles contained the ionizable lipid, DSPC, cholesterol and PEG-lipid (with PEG average molecular weight = 2000), and the PEG-lipid mole % in the formulation is either given or can be calculated. In addition, both the siRNA dose and the percentage knockdown or percentage gene expression resulting from a specific siRNA dose must be provided. Five papers were selected to prepare the data set (Jayaraman et al., 2012; Alabi et al., 2013; Kumar et al., 2014; Whitehead et al., 2014; Rajappan et al., 2020). Wherever the values for the gene expression or dose were not provided numerically, these values were obtained from the relative figures using WebPlotDigitizer v4.2. In case two or more lipids had the same 2D structure, one of them was retained. If an ionizable lipid lacked a well defined *in vivo* efficacy measure, such as a definite dose or knockdown %, it was omitted.

#### 2.1.2 Calculation of the 2D molecular descriptors

The structures of the ionizable lipids were drawn using ACD Chemsketch, and the structures were saved as either individual MDL.mol files or combined together into a single.sdf file using OpenBabel v2.4 (O'Boyle et al., 2011). The following software packages were used for the calculation of the 1D/2D molecular descriptors: Padel Descriptor v2.21 (Yap, 2011), RDKit 2017, and ToMoCoMD QuBiLS-MAS 2020 (Valdés-Martiní et al., 2017). For the calculation of the QuBiLS-MAS descriptors, the following settings were selected: linear algebraic form, atom-based, non-stochastic matrix form, and total groups.

#### 2.1.3 Data set preprocessing

The initial data set containing the descriptors was further processed by removing columns having one or more of either missing or not available (NA) entries. Columns with same-value entries were also removed. If certain columns in the data set showed a high correlation (cutoff r = 0.98) with each other (Racz et al., 2019), all the columns were removed except for one column which has the lowest average correlation with the other descriptor (predictor) columns in the data set. In addition, the formulation descriptor (PEG mol%) and percentage knockdown resulting from a specific siRNA dose were added as predictors. The data set descriptor columns were scaled by calculating the z-scores. The siRNA nanoparticles *in vivo* efficacy was included as the response variable; logarithm of the dose resulting in a specific knockdown percent.

### 2.2 Principal component analysis of data set

PCA of the scaled data set predictor columns (without response columns) was carried out using ChemometricsWithR package through R software v3.5.

### 2.3 Splitting the data set into training and validation sets

For modeling purposes, the data set entries were split into a training set (75% of entries) and a validation set (25% of entries). This process was carried out two times separately on the data set where the validation set entries (or observations) were selected either by random selection or by selecting sequentially every fourth entry in the set, with the remainder of the entries in the data set taken as the training set.

### 2.4 Machine learning models

The modeling process was carried out using either R software version 3.5 or Microsoft Open R v3.5. The following R packages were used for all modeling methods: caret (Deist et al., 2018) and Metrics (Hamner et al., 2018).

Artificial neural networks (ANNs) are a collection of linear and non-linear functions that map an input to an output. These functions can approximate a non-linear complex function. The idea behind the inner working of ANNs is that input data (**x**) are scaled and combined in a linear manner in the form of **Wx** + b, where W is the weights matrix and b is bias, and then the output of this linear combination is fed into a non-linear function (called activation function), the output of which could be used as an input to the next layer and/or to a final output layer (Wesolowski & Suchacz, 2019). For ANN modeling, nnet package was used. The hyperparameters were one hidden layer, two nodes and a weight decay of 0.1 for training and 0.001 for final validation set predictions.

Support vector machines (SVM) are a supervised machine learning technique. For classification, SVM aims to find a hyperplane (decision surface) that can separate two classes of observations with a maximum margin of separation (Maltarollo et al., 2019). Similarly, SVM regression follows the same logic of finding a hyperplane, but with a fixed margin width, epsilon (ε), within which the prediction error is considered zero, and the hyperplane found should minimize the sum of squared error. To enable the formulation of non-linear decision surfaces, a kernel function is applied. The general form of the kernel functions is K (**x1**,**x2**) = <φ(**x1**),φ(**x2**)>, where **x1** and **x2** are two data points. The kernel function thus avoids the actual calculation of the function φ (Heikamp & Bajorath, 2014). SVM regression modeling (epsilon-regression) was carried out using kernlab package (Karatzoglou et al., 2004), with epsilon value of 0.1 and the kernel chosen to be the Gaussian radial basis function kernel defined as $K\left(x,x_{i}\right)=-\sigma {\left|\left|x-x_{i}\right|\right|}^{2}$ , where σ is the inverse width parameter and is determined by the package’s sigest function.

Partial least squares (PLS) regression is another supervised learning technique (Hathout et al., 2020b). PLS combines dimensionality reduction of the data with a regression model. PLS formulation of the latent variables (scores or components) is carried out with the aim of maximizing the covariance of the components with the response variable, which differentiates PLS from regular principal component analysis (PCA) (Boulesteix & Strimmer, 2007). The response variable in PLS may be univariate or multivariate. For the prediction of a new data point response ${\hat{y}}_{o}^{\prime }$ from a predictor point $x_{o}^{\prime }$ , the following equation applies: ${\hat{y}}_{o}^{\prime }=\left(\frac{1}{n}\right)\sum_{i=1}^{n}y_{i}^{\prime }+B^{T}\left(x_{o}-\left(\frac{1}{n}\right)\sum_{i=1}^{n}x_{i}^{\prime }\right)$ . B is the matrix of regression coefficients, and is defined as: **B** = **W** (**T** ^T^ **T**)^−1^ **T** ^T^ **Y**, where **W** is the matrix of weights and **T** = **XW** (Boulesteix & Strimmer, 2007). PLS modeling was carried out using pls package (Mevik & Wehrens, 2007) with the number of principal components covering 98% of the variance.

### 2.5 Selection of the molecular descriptors by the evolutionary algorithm

An evolutionary algorithm was written as an R script to select the best descriptors for model building. 400 initial parent combinations of descriptors were randomly selected, and then each one of them was used as an input to construct the machine learning models that are used to predict the training set log (dose) values and their associated RMSEs (training RMSE).

The training RMSE is calculated as follows: the training set is split into three folds, two folds are used to construct the machine learning model, and the third fold is used as a test set to calculate training RMSE. After evaluating the training RMSE for all predictor combinations, the best combinations are kept as parents and are used to construct offspring combinations. The process is repeated until no further improvement in training RMSE for this specific test fold. The whole selection process is repeated for each of the remaining two test folds. The parameters for the evolutionary algorithm are as follows: population size 400, 25% elitism, 20% mutation, number of generations 10-20 and multipoint cross-over.

RMSE is calculated as: $RMSE=\sqrt{\left(\frac{\sum_{i=1}^{n}{\left(P_{i}-A_{i}\right)}^{2}}{n}\right)}$

Bias is calculated as: $Bias=P_{i}-A_{i}$ where **P_i_** and **A_i_** are the predicted and actual log (dose) values of observation (lipid or entry) *i* respectively, and *n* is the number of observations.

### 2.6 Ensemble learning by averaging of the validation set predictions

The best descriptor combinations that result in the lowest training RMSE were used as inputs for the machine learning modeling algorithm that was used in the training; either ANN, SVM or PLS regression. The central tendency of the validation set predictions were calculated as median of these values for each validation set lipid. The validation set RMSE (RMSE_val_) and coefficient of determination (R_val_^2^) were calculated using these median values. The R_val_^2^ is calculated as:

$$R_{val}^{2}=\frac{{\left(\sum_{i=1}^{n}\left(x_{i}-\bar{x}\right)\left(y_{i}-\bar{y}\right)\right)}^{2}}{\sum_{i=1}^{n}{\left(x_{i}-\bar{x}\right)}^{2}\sum_{i=1}^{n}{\left(y_{i}-\bar{y}\right)}^{2}}$$ (1)

where x_i_ and y_i_ are the i^th^ predicted (the median value) and actual responses respectively, $\bar{x}$ and $\bar{y}$ are the mean values of predicted and actual responses respectively.

### 2.7 Y-randomization of data set

To evaluate the validity of the resulting descriptor combinations, and the possibility that the obtained validation set predictions might be due to random chance, a Y-randomization of the training data set was carried out by randomizing the training set responses (Žuvela et al., 2015). The predictive models were then constructed by using these randomized responses for model training and subsequent validation as described in Section 2.6.

## 3 Results

### 3.1 Data set preprocessing and preparation

The number of observations included in the data set after omitting the lipids or entries that fit the omitting criteria explained in section 2.1.1 was 120 entries (rows). The resulting data set contained 438 predictor columns: 436 columns of molecular descriptors, and 2 columns for PEG mol% and knockdown %. In addition, one response column was included; logarithm of siRNA dose that results in a specific knockdown of the target gene. Table 1 provides summary of the data set.

**TABLE 1:** Summary of data set. The entries represent either distinct lipids or the same lipid but with different PEG mol% and/or knockdown %.

Index of entries	Number of entries per study	Reference
1-30	30	Rajappan et al. (2020)
31-62	32	Alabi et al. (2013)
63-95, 105	34	Jayaraman et al. (2012)
96-104	9	Kumar et al. (2014)
106-120	15	Whitehead et al. (2014)

### 3.2 Splitting the data set into training and validation sets

Two different methods were used to select the validation set entries, with the remainder of the entries in each splitting method being used for training the machine learning models. These selection processes resulted in the following data sets: training set1, validation set 1, training set 2 and validation set 2. These sets are shown in Table 2. Each training and validation set contained 90 and 30 entries, respectively.

**TABLE 2:** Training and validation sets 1 and 2.

Set	Training entries index	Validation entries index
**1**	3–6, 8–11, 13, 14, 18, 20, 21, 24–30, 33–37, 39, 43–49, 51, 54–60, 62–64, 66–69, 71–73, 75, 78–80, 82–84, 86, 89, 90, 92-108, 110–115, 117–120	1, 2, 7, 12, 15–17, 19, 22, 23, 31, 32, 38, 44, 50, 52, 53, 61, 65, 70, 74, 76, 77, 81, 85, 87, 88, 91, 109, 116
**2**	1–3, 5–7, 9–11, 13–15, 17-19, 21–23, 25–27, 29–31, 33–35, 37–39, 41–43, 45–47, 49–51, 53–55, 57–59, 61–63, 65–67, 69–71, 73–75, 77–79, 81–83, 85–87, 89–91, 93–95, 97–99, 101–103, 105–107, 109–111, 113–115, 117–119	4, 8, 12, 16, 20, 24, 28, 32, 36, 40, 44, 48, 52, 56, 60, 64, 68, 72, 76, 80, 84, 88, 92, 96, 100, 104, 108, 112, 116, 120

PCA is a dimensionality reduction method that transforms dataset features into a smaller number of new features called principal components. PCA scores are the weighted sums of the original features, and they represent the variance in the observations and can be used to detect similarities or dissimilarities among these observations.

**FIGURE 2:**
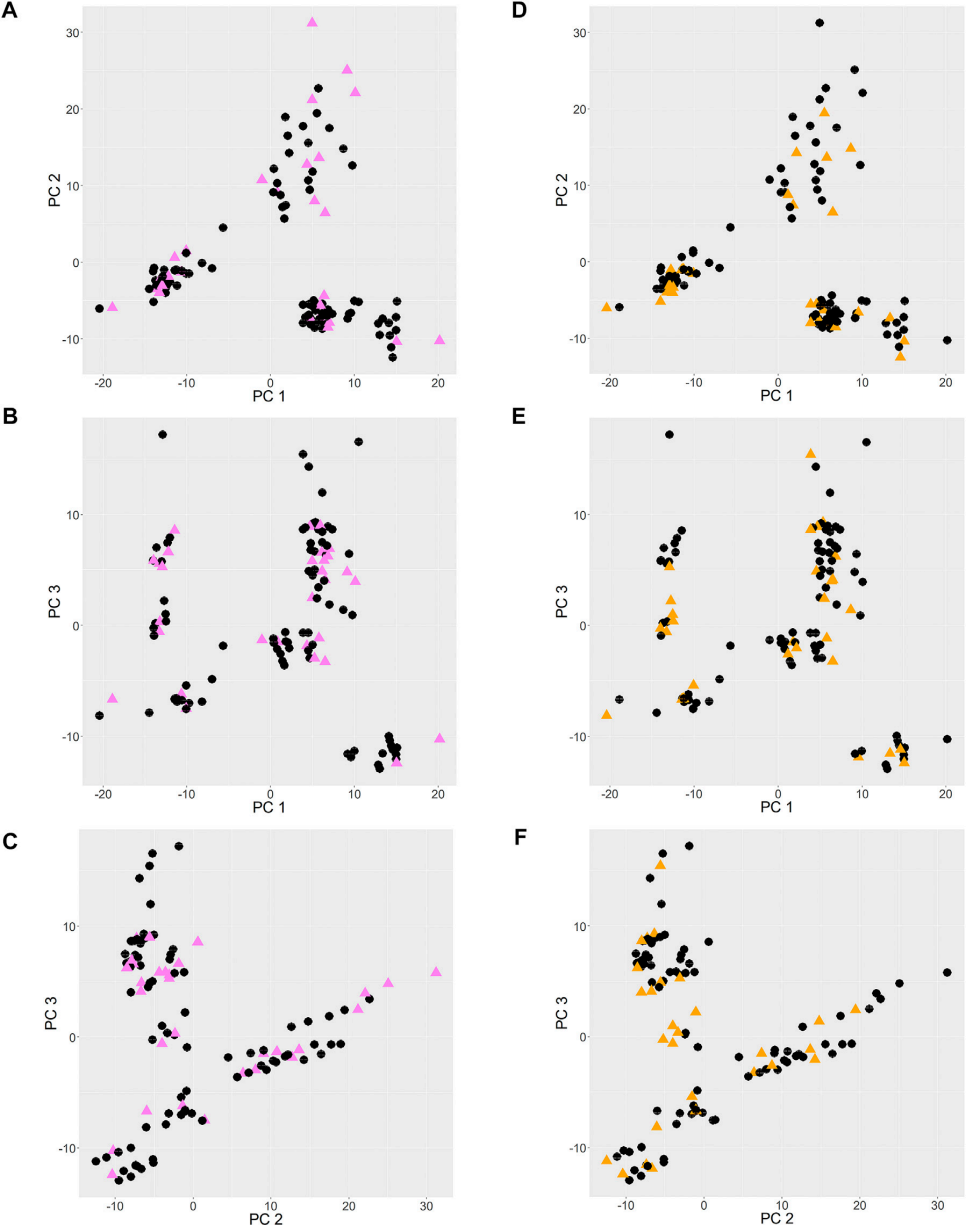
PCA score plots. **(A–C)**: training and validation set 1 entries are shown as black circles and pink triangles respectively. **(D–F)**: training and validation set 2 entries are shown as black circles and orange triangles respectively.

The PCA score plots are shown in Figure 2. Principal components 1, 2 and 3 (PC 1, PC 2 and PC 3) contributed to 22%, 19%, and 12% of the total variance, respectively. When points are near each other, this means that they represent observations that share similarities. The observations of validation set 1 and 2, shown as colored triangles, show uniform spread among those of training set 1 and 2 respectively, which infers that the training sets reasonably represent the characteristics of the validation sets.

### 3.3 Selection of the molecular descriptors by the evolutionary algorithm

**FIGURE 3:**
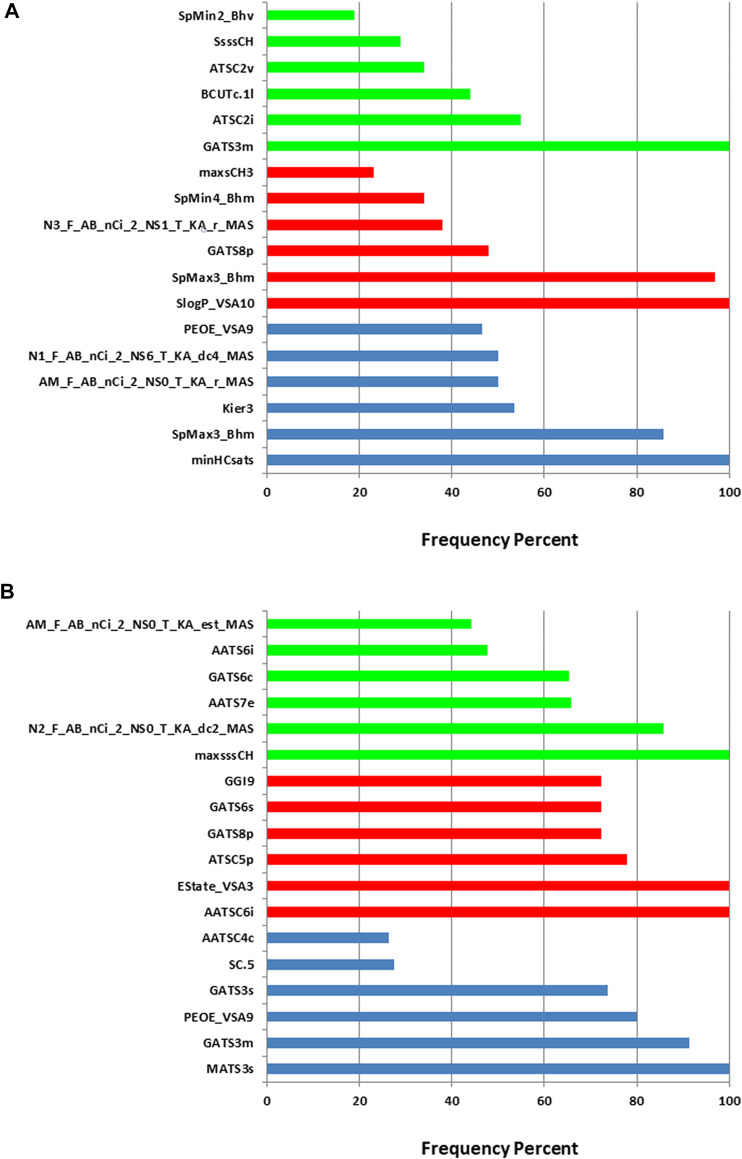
Relative frequencies of descriptors in the descriptor combinations selected by the evolutionary algorithm. **(A)** training set 1. **(B)** training set 2. Blue: ANN, red: SVM and green: PLS.

When constructing the descriptor combinations to be used as inputs for the machine learning algorithm, the PEG mol% and the knockdown % were always included in the combinations. Any additional molecular descriptors were added and selected by the evolutionary algorithm. Figure 3 shows the top six molecular descriptors with the highest frequencies of appearance in the descriptor combinations that are selected by the evolutionary algorithm. For each machine learning method, ANN, SVM or PLS, the descriptor with highest frequency was considered 100% and the other descriptors frequencies were calculated relative to it. It is evident that each machine learning model resulted in different top descriptors. It is also clear that the training sets one and two resulted in different top descriptors for the same machine learning method. The only common descriptors, taking the two training sets and the three machine learning methods in consideration, were PEOE_VSA9, GATS3m, and GATS8p. PEOE_VSA9 is a Van der Waals surface area descriptor that describes atomic partial charges. GATS3m and GATS8p are Geary autocorrelation - lag three weighted by atomic masses and Geary autocorrelation - lag 8 weighted by atomic polarizabilities respectively. It should be noted that these descriptors are present in combinations of descriptors (predictors) including the PEG mol% and the knockdown %, thus, their direct influence on the *in vivo* performance of the ionizable lipids should be limited to this context.

The number of molecular descriptors in each descriptor combination as selected by the evolutionary algorithm is listed in Table 3. It is to be noted that these molecular descriptors are present in each combination in addition to both PEG mol% and knockdown %, with the latter two being present in each predictor combination. It was noticed that there were repeated combinations in the final selected combinations, as omission of descriptors by the evolutionary algorithm results eventually in offspring combinations of the same descriptors.

**TABLE 3:** The minimum, maximum and median number of the molecular descriptors in the final predictor combinations for each training set and machine learning method.

Training set	Machine learning method	min	max	Median
1	ANN	2	7	5
1	SVM	3	7	4
1	PLS	3	7	3
2	ANN	4	9	5
2	SVM	4	9	6
2	PLS	4	9	6

The improvement in predictions of the validation set responses at the end of the evolutionary algorithm is shown in Table 4. The RMSE_val_ in the table are calculated as the first quartile of the RMSE of predictions using the initial 400 descriptor combinations and the final 400 descriptor combinations at the end of the evolutionary algorithm iterations. It is clear that there were improvement in the quality of individual predictions for both validation sets and for all methods as evident by the decrease in the RMSE_val_.

**TABLE 4:** Improvement of quality of individual validation set predictions by the evolutionary algorithm.

Validation set	Machine learning method	Initial first quartile RMSE_val_	Final first quartile RMSE_val_
1	ANN	0.41	0.33
1	SVM	0.40	0.31
1	PLS	0.41	0.29
2	ANN	0.40	0.35
2	SVM	0.39	0.36
2	PLS	0.44	0.37

The predictive performance of the machine learning models was evaluated by predicting the validation sets responses. The validation sets were neither used in the selection of best descriptor combinations by the evolutionary algorithm nor they were used in the training of the predictive models, thus, the validation sets represent external unkown test samples for the machine learning models. Using the descriptor combinations selected by the evolutionary algorithm, the median (averaged) predictions of the validation sets one and two resulted in R_val_^2^ of 0.72–0.89 and RMSE_val_ of 0.23–0.36 (Table 5). The machine learning method used to predict the validation set responses had a strong effect on the predictive performance, with the ANN predictions resulting in the highest R_val_^2^ of 0.89 and 0.80 for validation sets one and two respectively. Similarly, ANN resulted in the lowest RMSE_val_ of 0.23 and 0.30 for validation sets one and two respectively. There were also a difference in the predictive performance between validation sets one and two (Table 5), which reflects the effect of both the training set and validation sets compositions. Supplementary Figure S1 shows the structure of a model ANN, with one input layer, 2 nodes in the hidden layer, and one outcome node. The weights are also provided. To investigate if the ANN will perform better even if a different random sampling of training/validation sets was carried out, a third set (set 3) where validation lipids were selected randomly was prepared (Supplementary Table S1). The predictive performance of this set is presented in Supplementary Table S2 where the RMSE_val_ and R^2^ of ANN were better than those of SVM and PLS. Taken together, sequential sampling of validation lipids (set 2) as well as random sampling (set 1 and set 3) showed better performance for ANN.

**TABLE 5:** Evaluation of predictive performance of the different machine learning models.

Set	Machine learning model	RMSE_val_	R^2^_val_
1	ANN	0.23	0.89
1	SVM	0.32	0.81
1	PLS	0.26	0.86
2	ANN	0.30	0.80
2	SVM	0.36	0.72
2	PLS	0.34	0.75

### 3.4 Evaluation of predictive performance by predicting validation set responses

**FIGURE 4:**
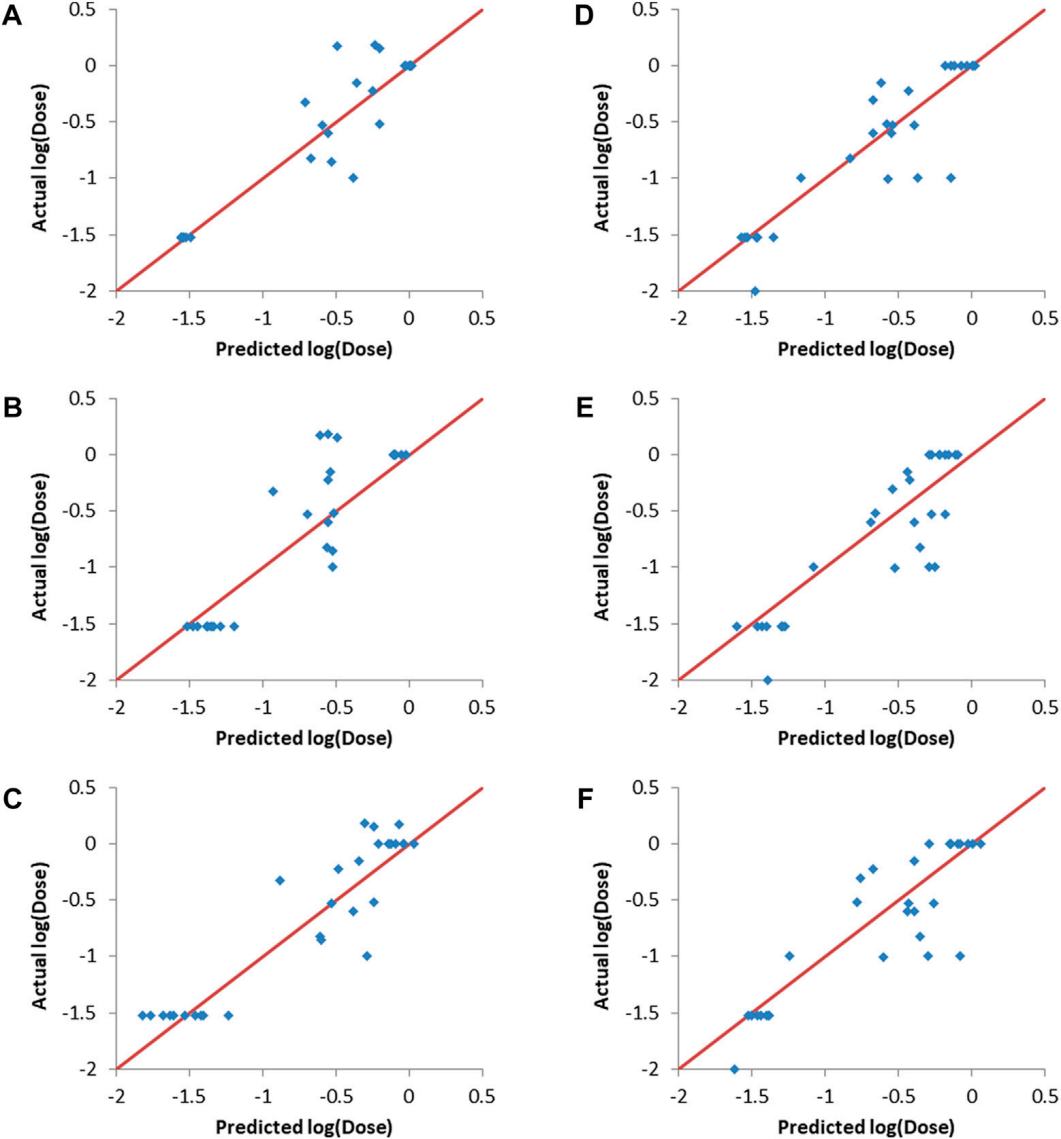
Actual vs predicted log (dose) plots. **(A–C)**: Validation set 1, **(A)** ANN, **(B)** SVM and **(C)** PLS. **(D–F)**: Validation set 2, **(D)** ANN, **(E)** SVM and **(F)** PLS.

Figure 4 shows that the three machine learning methods resulted in good validation sets predcitions, as evident from the predicted points being close to the straight lines (shown in red and representing perfect correlation) in the actual vs predicted plots. It is also clear that the different machine learning models were capable of differentiating between the lipids (entries) with low log (dose), which are the desirable lipids (or formulations), and the lipids/formulations with higher doses.

The curated scaled data set together with an example of the resulting predictor combinations (training set 1) after selection by the evolutionary algorithm and ANN is provided as Supplementary Materials. An R script for calculating the median predictions of validation set 1 and the assocciated R_val_^2^ and RMSE_val_ using the data set and the descriptor combinations is also provided as Supplementary Material.

### 3.5 Y-randomization of training set responses

**FIGURE 5:**
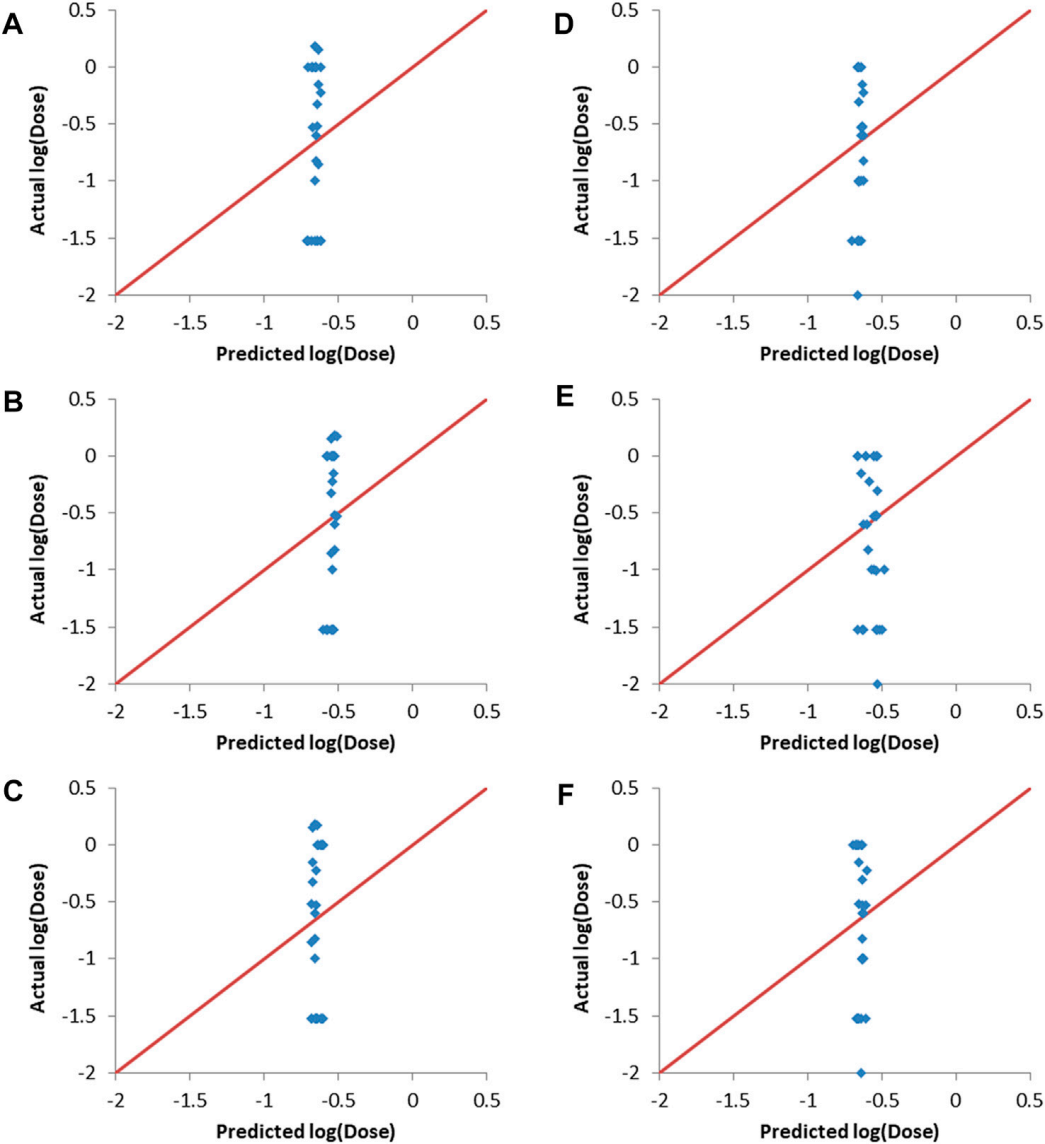
Actual vs predicted responses of validation sets after Y-randomization of training sets responses. **(A–C)** validation set one. **(A)** ANN, **(B)** SVM and **(C)** PLS. **(D–F)** validation set two. **(D)** ANN, **(E)** SVM and **(F)** PLS.

Y-randomization involves randomizing the responses column and then training the predictive models using one of the machine learning methods, with the input descriptors and the responses being mismatched due to the randomization of the responses (Rucker et al., 2007). Y-randomization was carried-out using the final combinations selected by the evolutionary algorithm as inputs. The resulting predictions together with the actual responses are shown in Figure 5. It can be seen that there is no correlation between the predicted and actual responses for both validation sets and for all of the machine learning methods used. The R_val_^2^ values ranged from 0.014 to 0.116, with RMSE_val_ values between 0.66 and 0.68. This lack of correlation proves that the results obtained without randomization of the responses (Figure 4 and Table 5) where not due to random chance.

### 3.6 Effect of setting the formulation descriptor PEG mol% to either the maximum or the minimum value

To examine if the predictive models capture the changes in the formulation descriptor; the PEG mol%, the values of this descriptor were set to either its maximum value or rather its minimum counterpart. It is well known that when using siRNA lipoplexes, there is a certain PEG mol% that results in the maximum *in vivo* efficacy in addition to stabilization of the nanoparticles (Mui et al., 2013; Kumar et al., 2014; Sakurai et al., 2020). The general trend is that increasing the PEG mol% more than a specific mole percent results in decreasing the *in vivo* efficacy. It is generally found that PEG mol% that is equal to 10 decreases efficacy, while values around 1.5% results in good *in vivo* efficacy (Jayaraman et al., 2012; Kumar et al., 2014). Hypothetically, it is assumed that if the PEG mol% descriptor values were set to the maximum (equivalent to 10%), the *in vivo* efficacy should decrease, i.e., the log (dose) should increase. On the other hand, if the PEG mol% values are set to the minimum (equivalent to 1.5%), then the *in vivo* efficacy should generally improve for the validation sets lipids that have PEG mol% higher than 1.5%.

**FIGURE 6:**
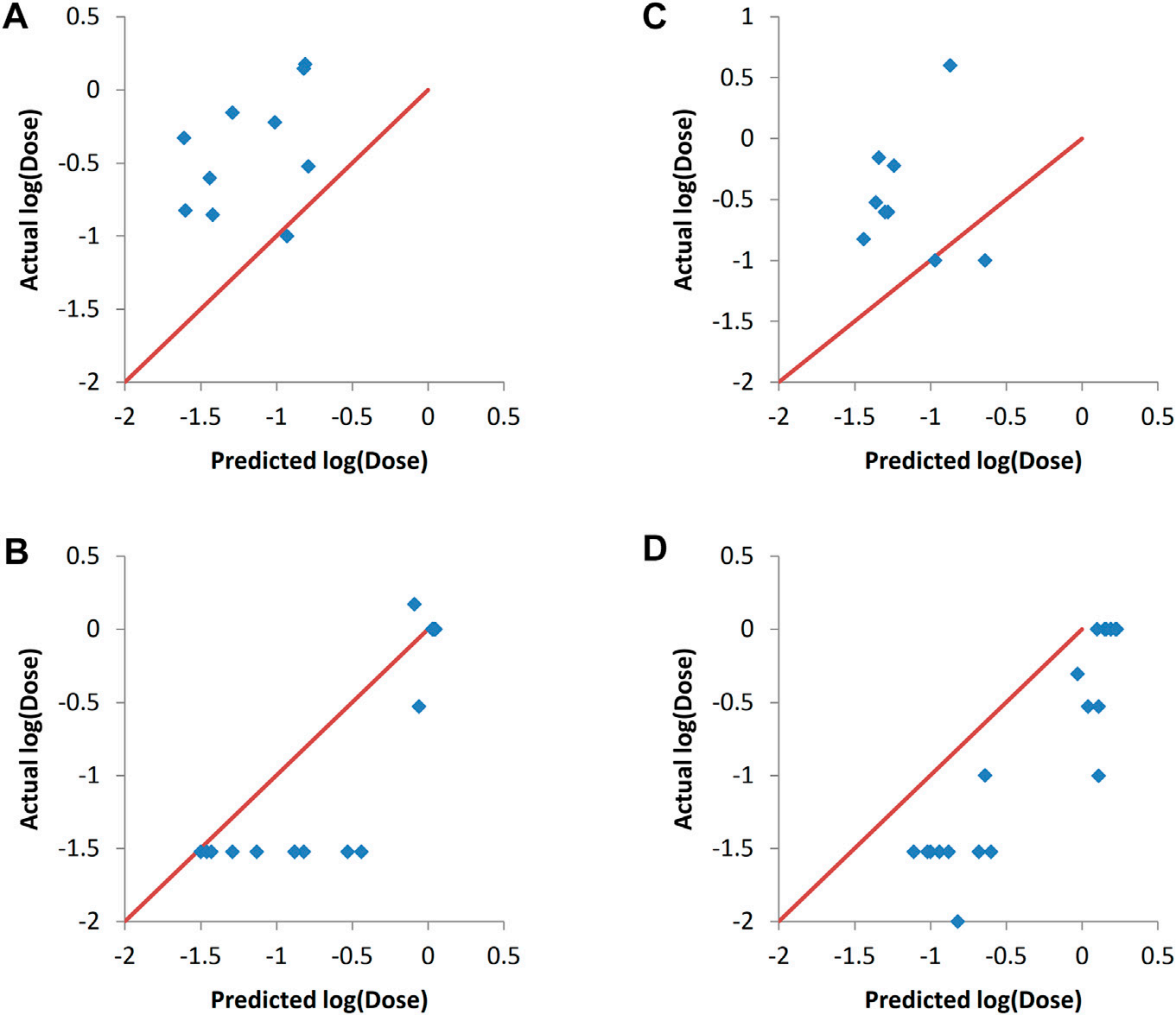
Actual vs predicted responses of validation sets after setting the values of the PEG mol% descriptor to either the minimum value **(A, C)** or the maximum value **(B, D)**. The modeling was carried out by ANN. **(A)** and **(B)** validation set one. **(C)** and **(D)** validation set 2. The validation sets entries with the actual PEG mol% being the minimum value were omitted from A and C, while those with the actual PEG mol% being the maximum were omitted from B and D for visualization clarity.

It can be seen in Figures 6A,C that setting the PEG mol% to the minimum values resulted in a decrease in log (dose) as expected, as evident by the shift of the predictions towards the left hand side. Similarly, setting PEG mol% to the maximum value resulted in shifting of the predicted log (dose) towards higher values as it would be expected (Figures 6B,D). These results prove that the predictive models could capture the significance of the formulation descriptor in a correct manner. ANN was the method used to train the models because it resulted in the best predictions as shown in Figure 4 and Table 5. Similar results were obtained with SVM and PLS regression (data not shown).

### 3.7 Refining the predictions by determining the applicability domain

**FIGURE 7:**
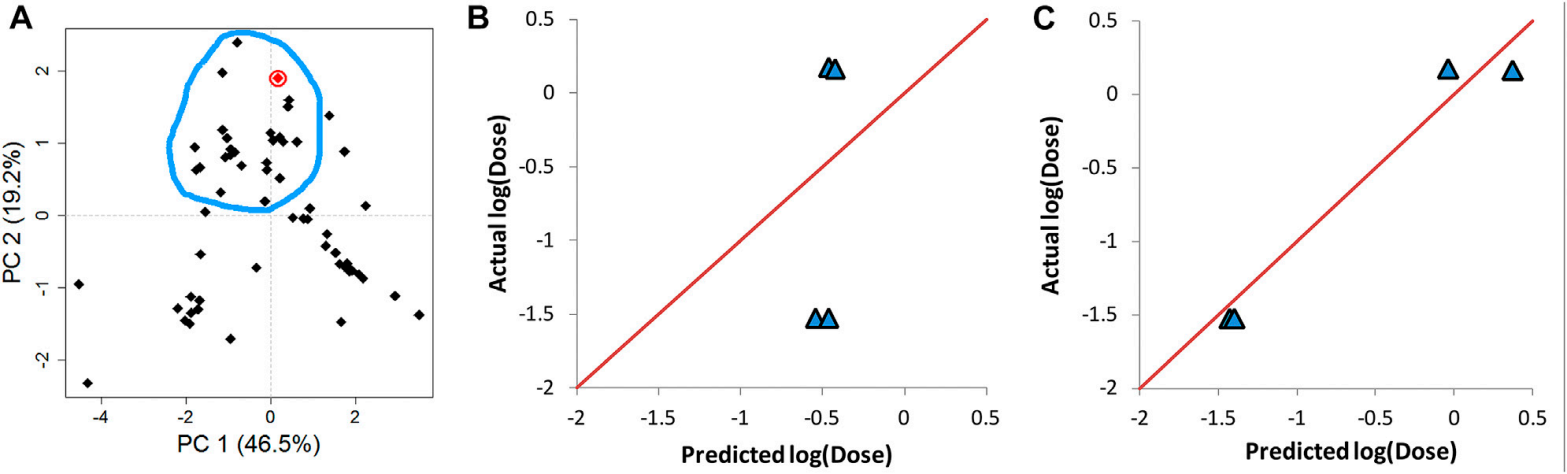
Determination of the applicability domain (AD) of four lipids from validation set one. **(A)** PCA of training set together with one of the validation set lipids (lipid 15) shown in red circle. **(B)** The actual vs predicted plot before determining AD. **(C)** The actual vs predicted plot after determining AD. Predictions in C and B are carried out by ANN. The red line in B and C represents perfect correlation between actual and predicted values.

AD represents a theoretical region in the chemical space of the training set samples. It is expected that predicting the response of unknown samples, e.g., an external validation set, results in more reliable predictions when the unknown samples fall within this region (Weaver & Gleeson, 2008; Tropsha, 2010). One method to determine this region is by applying PCA on the training and validation data, and constructing the region of applicability accordingly (Weaver & Gleeson, 2008). Figure 7A shows the score plot of one fold of training set one and lipid 15 which belongs to validation set one (shown as a red circle). The descriptors combination used to perform PCA were chosen randomly from one of the final combinations selected by the evolutionary algorithm. The region encircled by the blue line is the AD, and it was determined manually by excluding from the training entries under consideration those which are far from lipid 15 in the space generated by plotting PC 1 and PC 2. The first two components capture 66% of the variance in the data. The training lipids selected within the AD were then used by ANN to predict the response of lipid 15. This procedure was repeated for another three lipids from the same validation set. The four lipids selected were chosen based on them having the highest biases in their predicted values (Table 6). It is clear by comparing the predicted responses in Table 6 before and after carrying out the selection of training lipids lying in the AD that there was a vast improvement in the quality of the predictions as seen from the much lower bias values before and after selection. In addition, the R^2^ for the four lipids was 0.47 and 0.96 before and after applying AD lipid selection respectively, showing significant improvement in the prediction accuracy of these lipids. The impact of improvement of predictions can be seen in Figures 7B,C, where the predictions lie much closer to the red line in Figure 7C compared to 7B. Since this procedure is carried out manually, we suggest that is should be performed as a refining step for the set of lipids that will be chosen for further wet lab experimentations.

**TABLE 6:** Refinement of predictions by selecting training lipids within AD.

Lipid index	Actual response Log (dose)	Predicted response before applying AD	Predicted response after applying AD	Bias before applying AD selection	Bias after applying AD selection
15	−1.52	−0.55	−1.43	0.97	0.09
16	−1.52	−0.47	−1.40	1.05	0.12
70	0.18	−0.47	−0.04	−0.65	−0.22
109	0.17	−0.43	0.37	−0.60	0.20

## 4 Discussion

This study provides a computational framework to predict *in silico* the *in vivo* performance of the siRNA lipid nanoparticles. The main question answered in this manuscript is how to predict the siRNA dose of siRNA lipid nanoparticles given a set of molecular descriptors, formulation characteristics and a required knockdown percent. From the results presented in this work, it is evident that this objective was successfully achieved. In order to produce high quality predictions, the following aspects were carefully considered; 1) The selection of the optimal descriptor combinations 2) The modeling approach 3) Validation of the machine learning models using external validation sets and 4) Improving the predictive outcome of the final models by selecting the training set lipids according to the applicability domain.

When preparing the data set, 2D descriptors were calculated from the ionizable lipid structures rather than 3D descriptors. The reason for avoiding the use of 3D descriptors is that not all the lipids were defined in terms of their stereochemistry. In addition, the optimized 3D structure of a single molecule present in the solution state might differ from the 3D structure of the same molecule if present in close contact with other molecules as in the case of nanoparticles. The effect of the source of the 3D structure and its preparation method and energy minimization in relation to the quality of predictions of three classes of molecules (anilines, carboxylic acids and phenols) has been previously shown (Geidl et al., 2015). There are other potentially important formulation factors that may play a role in the modeling, e.g., particle size and siRNA to lipid ratio, however, they were not included as they were not reported consistently in the selected literature. For example, particle size was reported on occasions as a wide range instead of well defined values. Nanoparticles pK_a_ was also not included in the descriptors as it is not initially a controllable variable that could be pre-determined compared to the formulation parameters, the lipid structure (by its design) and the required percent knock-down.

As for the descriptor selection, an evolutionary algorithm was used. The evolutionary algorithm comprised: (a) “selection” of the descriptor combinations based on an optimization criterion; the RMSE of the test set after splitting the training set into three folds during training, (b) “crossover” of the selected parent combinations to make new offspring combinations and (c) “mutations” of certain descriptors in offspring combinations. These processes are main elements in any evolutionary algorithm (Sipper et al., 2018). Evolutionary algorithms are suitable for solving the problem of finding optimized solutions of combinations from a set of inputs (descriptors in this case) where an exhaustive search that covers all possible combinations is computationally not feasible (Douguet et al., 2000). In addition, evolutionary algorithms perform better in the presence of noise in data (Arnold & Beyer, 2003). They also offer a set of solutions, which allows for averaging of the predictions of these solutions to get a better predictive performance.

Accordingly, evolutionary algorithms and their variants, such as genetic algorithms, were used to refine the structure of Au nanoparticles (Yu et al., 2016) and to optimize descriptor combinations in counter-propagation artificial neural networks models used to classify drugs as being either hepatotoxic or non-hepatotoxic (Bajželj & Drgan, 2020).

The R software or Microsoft Open R as well as the cheminformatic packages used in this study are available for free, which makes them completely accessible for a wider population of researchers. Using free modeling tools is gaining momentum, for example, additional web-accessible prediction tools and machine-learning based algorithms were successfully utilized to design amphiphilic peptide scaffolds for engineering drug delivery nanoassemblies (Feger et al., 2020).

The modeling approach in the current work involved three machine learning methods: ANN, SVM and PLS. These methods differ in their inner workings. The ANNs are considered a collection of linear and non-linear functions that are governed by the choice of the ANN architecture and activation functions. The SVM belongs to the class of kernel algorithms while PLS regression depends on the construction of latent components (principal components) that result in the best covariance with the response variable. Thus, the difference in their predictive performance could be expected. In order to improve the predictive outcome of the final models, averaging of the predicted response values was carried out. Averaging of predictions belongs to a set of machine learning methods called ensemble learning, and usually results in better prediction outcome (Oprisiu et al., 2012).

Machine learning models require reliable validation to be sure about their ability to successfully predict unknown observations responses. For this purpose, many metrics were suggested and used such as R^2^, Q^2^ and external validation set R^2^. Similarly, RMSE of training set predictions, cross-validation RMSE and external validation RMSE are used for the same purpose. In addition, techniques such as Y-randomization are used to exclude the possibility of the model predictions being due to random chance. Q^2^, the cross-validation coefficient of determination, does not necessarily correlate with good predictive performance for external validation sets (Golbraikh & Tropsha, 2002). Thus, in this work the validation of the final machine learning models was carried out by predicting responses of two external validation sets as well as performing Y-randomization of training set responses, conforming to the best model validation practices (Tropsha, 2010; Maleki et al., 2020). The results showed that the obtained models are reliable.

It is suggested that training set composition and/or the relevant properties of the validation set in relation to the training set governs the predictive performance (Martin et al., 2012; Nalepa & Kawulok, 2019). One way to overcome this is to make sure that the validation set observations are within the applicability domain of the training set (Tropsha, 2010; Maleki et al., 2020). In the current work, rather than selecting the validation set observations that lie within the training set applicability domain, a reverse approach was followed; a subset of the training set elements was selected to be close in the predictor space to the validation element under investigation, i.e., these selected training set elements were used to construct the applicability domain. PCA of the training set and the validation set lipid was carried out to determine this applicability domain visually (Figure 7A). It is evident from the results presented in Figures 7B,C and Table 6 that this protocol resulted in significant improvement in performance.

Recently, *in vitro* cellular uptake of siRNA nanoparticles formulated with hydrophobic derivatives of polyethyleneimine (PEI) was predicted by QSAR modeling using either linear regression, random forests or multilayer perceptron, with the non-linear methods proving to be more efficient than linear regression (Nademi et al., 2021). The R^2^ of the external test set ranged between 0.34 and 0.50 depending on the machine learning method used and on the number of input descriptors, with the initial number of 26 descriptors being reduced either by binary encoding or by backward elimination.

Overall, in the current work, *in vivo* performance of siRNA nanoparticles could be predicted accurately by combining machine learning techniques with cheminformatics. This framework will greatly enhance the development of siRNA nanomedicines.

## 5 Conclusion

The *in vivo* efficacy of siRNA ionizable lipid nanoparticles could be predicted with excellent accuracy provided careful modeling choices. Calculating molecular descriptors of a series of ionizable lipids followed by selecting best descriptor combinations using an evolutionary algorithm in combination with machine learning modeling by ANN, SVM and PLS and then finally making an ensemble of the predictions by calculating the median of validation set predictions resulted in successful predictions of *in vivo* activity of siRNA ionizable lipids nanoparticles. Depending on the machine learning method and the validation set, R_val_^2^ of up to 0.89 could be achieved. Further improvement of validation set entries with high bias was achievable by selecting training lipids within the applicability domain, with R_val_^2^ improvement from 0.47 to 0.96.

This *in silico* approach allows the evaluation of virtually an endless number of ionizable lipids prior to their actual synthesis and wet lab evaluation and hence saving valuable resources and time while exploring the vast chemical space of these lipids and their formulations.

## Data Availability

The original contributions presented in the study are included in the article/Supplementary Material, further inquiries can be directed to the corresponding author.
