# Supplementary material for: In silico prediction of siRNA ionizable-lipid nanoparticles In vivo efficacy: Machine learning modeling based on formulation and molecular descriptors
Source: Front Mol Biosci. 2022 Dec 21;9:1042720. doi: 10.3389/fmolb.2022.1042720 (PMC9811823; doi:10.3389/fmolb.2022.1042720)
Supplement: Supplementary file 1 [file DataSheet2.pdf]

Supplementary Figure 1. Structure of a 7:2:1 ANN model. I1 to I7 are input layers variables. B1: Bias of hidden layer. H1 and H2 are hidden layer two nodes. B2: Bias of output layer. O1: Outcome node. Lines in grey colour indicate negative weights while black lines indicate positive weights. The thickness of the line corresponds to the relative magnitude of the coefficients.

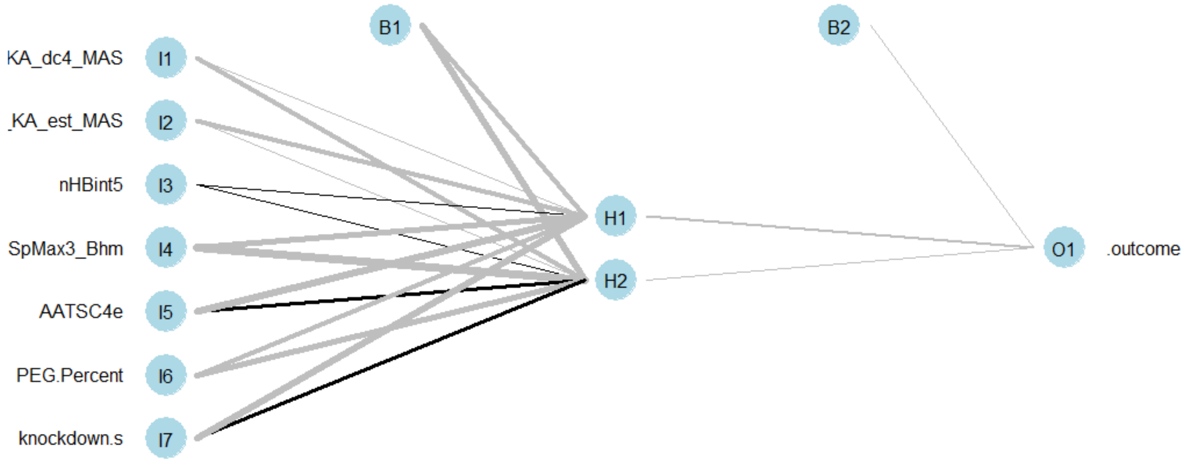

The weights of ANN shown in supplementary figure 1 are:

Input layer to node 1 in hidden layer:

-3.7252383, -0.9513748, -4.4572328, 0.7992806, -5.4035883, -6.0521087, -4.4866610, -6.3173371

Input layer to node 2 in hidden layer:

-7.1024413, -3.6315459, -0.1505515, 0.8427155, -7.1626434, 2.9634617, -5.2756651, 2.4894205

Hidden layer to output layer:

-0.09611705, -1.42868251, -0.83603192

Supplementary Table 1. Training and validation set 3.

Set	Training entries index	Validation entries index
**3**	1, 2, 4, 5, 7-10, 12-16, 19, 21-28, 30, 31, 33-36, 38, 41-43, 45-47, 49, 50, 52-54, 57-59, 61-70, 72, 74, 75, 77-79, 81, 83-85, 87-92, 94, 95, 97-99, 101-107, 109-116, 118	3, 6, 11, 17, 18, 20, 29, 32, 37, 39, 40, 44, 48, 51, 55, 56, 60, 71, 73, 76, 80, 82, 86, 93, 96, 100, 108, 117, 119, 120

Supplementary Table 2. Evaluation of the predictive performance of different machine learning methods for set 3 where train and validation samples were selected randomly.

Set	Machine learning Model	$RMSE_{val}$	R^2_{val}
3	ANN	0.27	0.85
3	SVM	0.35	0.75
3	PLS	0.36	0.74
