# Supplementary figures and images for: In silico prediction of siRNA ionizable-lipid nanoparticles In vivo efficacy: Machine learning modeling based on formulation and molecular descriptors
Source: Front Mol Biosci. 2022 Dec 21;9:1042720. doi: 10.3389/fmolb.2022.1042720 (PMC9811823; doi:10.3389/fmolb.2022.1042720)

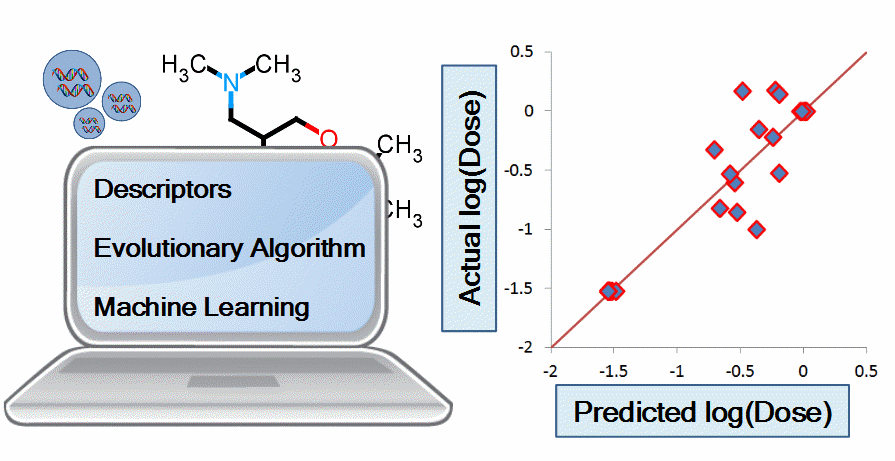

Supplement: Supplementary file 3 [file Image1.TIF]
